# Managing HIV-Associated Hodgkin Lymphoma During the COVID-19 Pandemic: Case Report and Literature Review

**DOI:** 10.3390/v17030404

**Published:** 2025-03-12

**Authors:** Monica-Daniela Padurariu-Covit, Mihaela Andreescu, Elena Niculet, Alina Plesea-Condratovici, Manuela Arbune

**Affiliations:** 1 Doctoral School of Biomedical Sciences, Dunarea de Jos University of Galati, 800008 Galati, Romania; monica.padurariu@ugal.ro; 2 Hematology Department, Emergency County Hospital Sf. Apostol Andrei, 800578 Galati, Romania; 3 Faculty of Medicine, Titu Maiorescu University, 031593 Bucharest, Romania; tevetmihaela@gmail.com; 4 Hematology Department, Colentina Clinical Hospital, 020125 Bucharest, Romania; 5 Morphological and Functional Sciences Department, Faculty of Medicine and Pharmacy, Dunarea de Jos University of Galati, 800008 Galati, Romania; helena_badiu@yahoo.com; 6 Morphological Department, Emergency County Hospital Sf. Apostol Andrei, 800578 Galati, Romania; 7 Medical Department, Medicine and Pharmacy Faculty, Dunarea de Jos University of Galati, 800008 Galati, Romania; 8 Clinical Medical Department, Dunarea de Jos University of Galati, 800008 Galati, Romania; manuela.arbune@ugal.ro; 9 Infectious Diseases Clinic Hospital Sf. Cuv. Parascheva, 800179 Galati, Romania

**Keywords:** HIV, Hodgkin lymphoma, COVID-19, late presenter, acquired immunodeficiency

## Abstract

The COVID-19 pandemic delayed the consultation of many patients with specialists. We present the case of a 57-year-old patient with HIV infection, COVID-19 pneumonia, and Hodgkin lymphoma. Discordant immunohistochemistry results from biopsy samples delayed the hematological diagnosis and initiation of oncological therapy. The late diagnosis of HIV infection at the stage of severe immunosuppression, along with advanced Hodgkin lymphoma and COVID-19 co-infection, represents a complex pathogenic triad that is challenging to manage. Healthcare-associated COVID-19 infections pose a significant risk during the pandemic for chronic patients requiring frequent hospital visits.

## 1. Introduction

COVID-19 had a major impact on the healthcare routines of medical facilities in the first two years of the pandemic. Most countries of the world adopted measures to reduce the risk of overloading the healthcare system and to limit healthcare transmission, including postponing non-urgent clinical visits, canceling elective surgeries, increasing intensive care capacities, and restricting social life through national lockdowns [1]. Although these actions were effective in reducing the number of new COVID-19 infections, they may have also had a deleterious impact on the diagnosis of other major medical conditions [2].

The COVID-19 pandemic delayed the consultation of many symptomatic patients for medical care, resulting in delayed diagnoses and increased mortality from stroke, acute heart failure, pulmonary embolism, cancer, HIV infection, or chronic hepatitis [3,4,5,6,7]. The pandemic also caused setbacks in human immunodeficiency virus (HIV) testing, adding to other existing barriers such as fear, discrimination, or low-risk perception [7,8].

Our study highlights the interaction between COVID-19, HIV/AIDS, and Hodgkin’s lymphoma (HL), illustrated by a real case presentation and analysis of the medical literature, providing useful insights for medical practitioners in managing such complex cases.

## 2. Case Presentation

### 2.1. Demographic Data

We present the case of a 57-year-old Caucasian male, with secondary-level formal education, who was widower from a rural area, unemployed. He was a heavy smoker, consumed alcohol occasionally, and engaged in unprotected sexual relationships. His medical history included hypertension.

### 2.2. Medical History

In June 2019, he was hospitalized in the pulmonology department due to a dry cough and fever. A thoracic computed tomography (CT) scan detected two pseudonodular lesions in the right lung, multiple thoracic adenopathies, and an adenomatous node in the left adrenal gland. He was referred to the Thoracic Surgery Department for a biopsy of a right axillary lymph node. The anatomopathological examination was suggestive of lymphoma; however, the diagnosis was invalidated by discordant immunohistochemistry tests, which characterized the condition as reactive lymphadenitis. Notably, CT imaging showed that the initially described lesions had decreased in size compared to an examination three months earlier. He was lost to follow-up from 2019 to 2021, including more than a year during the COVID-19 pandemic. Notably, the patient was not tested for HIV until 2021, despite having several risk factors and symptoms suggestive of the disease.

### 2.3. Clinical Presentation and Evaluation

The patient was admitted to the Hematology Department in May 2021, presenting with asthenia, loss of appetite, fever, profuse sweating, and weight loss exceeding 10 kg. The complaints have worsened especially in the past six months. The patient had not been vaccinated for COVID-19 prior to admission.

The clinical examination at admission revealed a body temperature of 38 °C, BP = 170/80 mmHg, HR = 102/min, RR = 16 b/min, SO_2_ = 98% (on air room), GSC = 15, but altered general condition, oral thrush, edema of the lower limbs, mild jaundice, enlargement of the liver and spleen, and generalized peripheral lymphadenopathy, with a maximum diameter of 4 cm in the right axilla. We considered the primary probability of a malignant disease diagnosis, as the medical history and clinical examination met seven out of eight risk factors according to Gaddey’s criteria (Caucasian, age over 40 years, male sex, lymphadenopathy lasting more than 4–6 weeks, lymphadenopathy involving at least two anatomical regions, and systemic signs). The only absent criterion was supraclavicular lymphadenopathy [9]. According to the Eastern Cooperative Oncology Group, clinical examination revealed an ECOG Performance Status Scale of 3–4 [10,11].

### 2.4. Diagnostic Investigations

Routine RT-PCR for SARS-CoV-2 was negative at admission.

Laboratory tests revealed severe pancytopenia, cholestasis, hypoproteinemia, and hypoalbuminemia (Table A1). Examination of the bone marrow aspiration, correlated with the complete blood count (CBC) and peripheral blood smear, suggested abnormal cellularity that could be associated with infectious, inflammatory, neoplastic, or drug-related conditions. Findings included erythroid, granulocyte, and platelet precursors with a moderate dysplastic appearance, but no macrophages were described. Tumor markers were normal, and a new lymph node biopsy was performed. An HIV test was positive, and in the second week, the patient was transferred to the Infectious Diseases Department for supplementary investigations. In addition, a repeat SARS-CoV-2 test was positive, as a consequence of a hospital-associated infection, although there were no additional specific signs of COVID-19.

ELISA and Western blot tests confirmed HIV infection. ARN-HIV was not available, but the low CD4-lymphocite count (CD4) of 112 cell/µL indicated severe immunosuppression. Tests for HBsAg, HBcAb, HCV-Ab, and syphilis were negative.

Respiratory function seemed normal, although the patient had a productive cough. The sputum culture was positive for *Candida* spp. and *Klebsiella pneumoniae*, possibly indicating that colonization exacerbated in the context of COVID-19. Multiple poorly defined opaque images in both lungs, mostly perihilar and fibrotic bands, were described in the X-ray. The differential diagnoses primarily considered included COVID-19 pneumonia, Pneumocystis jirovecii pneumonia, tuberculosis (TB), and lymphoma. Bronchoscopy and alveolar lavage or biopsy were not available in the COVID-19 context. TB investigations by GenXpert, Ziehl–Neelsen staining, and Löwenstein–Jensen cultures of the bone marrow aspirate and sputum were negative. A sputum sample examination with Toluidine Blue O stain was also negative for *Pneumocystis*.

**Figure 1:**
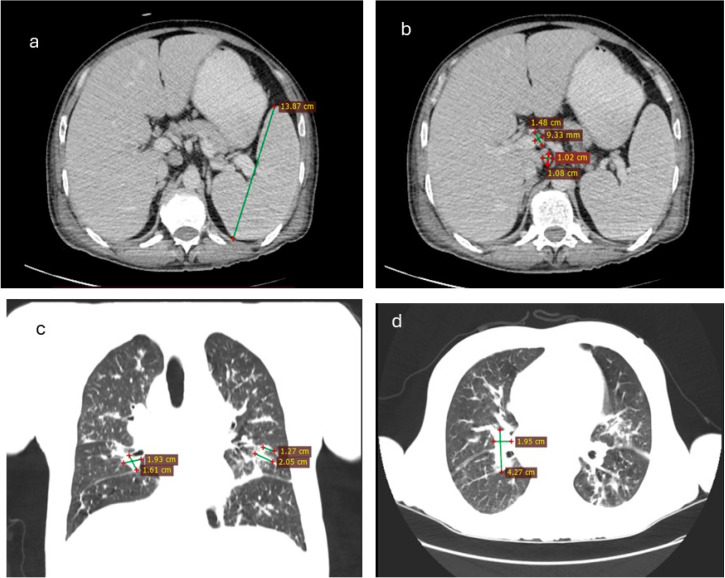
CT scanner particularities: (**a**) splenomegaly; (**b**) adenopathies in the liver hill; (**c**) fibrosis bands in the lungs (axial); (**d**) fibrosis bands in the lungs (sagital).

A new CT examination described splenomegaly measuring 140 × 97 mm, an enlarged right liver lobe measuring 203 mm, supra- and subdiaphragmatic adenopathy with a maximum diameter of 40 × 25 mm, new multiple lymph nodes in the hepatic hilum, celiomesenteric, retroperitoneal, and iliac regions with a maximum diameter of 14 × 9 mm, and the appearance of ascitic fluid localized in the paracolic and pelvic areas (Figure 1a,b). Lung imaging showed multiple bilateral “ground glass” infiltrates, micronodules, and fibrotic bands, which could be related to multiple etiologies with variable pathogenic mechanisms: partial filling of air spaces or collapse of alveoli, interstitial thickening, inflammation, edema, fibrosis, or lepidic proliferation of neoplasm (Figure 1c,d).

### 2.5. Therapeutic Interventions and Outcome

Early intervention with monoclonal antibodies for SARS-CoV-2 was not available. According to COVID-19 protocols, the patient was isolated in a dedicated department and received antiviral treatment (Remdesivir), antibiotics (Ceftriaxone, Co-trimoxazole), antifungal treatment (Fluconazole), corticosteroids, and a prophylactic dose of low-molecular-weight heparin. Hematological support included the transfusion of erythrocyte mass and platelet products, as well as the administration of granulocyte colony-stimulating factor.

Prior to the initiation of antiretroviral therapy, liver enzyme values showed moderate elevations, with aspartate aminotransferase (AST) at 79 U/L (normal range: 10–40 U/L) and alanine aminotransferase (ALT) at 84 U/L (normal range: 7–56 U/L), consistent with cholestasis.

Antiretroviral combination therapy with darunavir/cobicistat/emtricitabine/tenofovir alafenamide was initiated within the first two weeks following the HIV diagnosis. However, the patient experienced immunological failure, as evidenced by a decrease in CD4 count from 112 to 21/mm^3^. The laboratory test dynamics revealed progressive pancytopenia and cholestasis, along with variations in cytolytic markers and hypercoagulation (Table A1).

The patient subsequently died due to gastric hemorrhage and liver failure before any specific treatment for lymphoma could be administered. Notably, the RT-PCR test for SARS-CoV-2 remained positive for over two months until the patient’s death. A necropsy was not performed.

**Figure 2:**
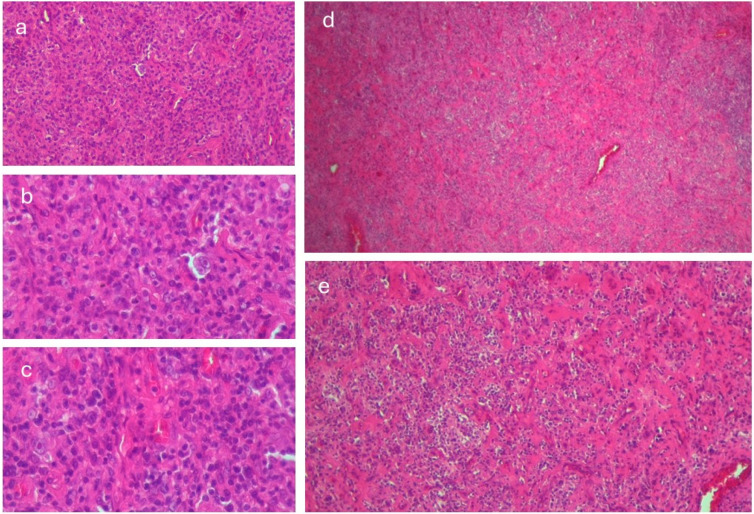
Histology of the lymph node: (**a**) Reed–Sternberg cell within an inflammatory background and narrow band fibrosis, hematoxylin and eosin (H&E), 200× magnification; (**b**) high-power view of a Reed–Sternberg cell, H&E, 400× magnification; (**c**) mononuclear Hodgkin cell, H&E, 400× magnification; (**d**) lymph node architectural effacement with narrow fibrotic bands, H&E, 40× magnification; (**e**) mild fibrosis with a mixed inflammatory infiltrate, H&E, 100× magnification.

Two weeks post-mortem, histopathological and immunohistochemical analysis of the lymph node was completed. Large cells exhibited CD30 positivity, while rare cells were CD20-positive and showed a high Ki-67 proliferation index, with negative staining for CD3. CD3 positivity was observed in typical small lymphocytes and activated T cells, one of which was PD-1-positive. The large cells also expressed PAX5 and were negative for CD15, supporting the diagnosis of mixed-cellularity classical HL (Figure 2).

Reconsidering the case, the final diagnosis was as follows: Multisystem organ failure consequent to COVID-19. AIDS late presenter. Classical HL (Ann Arbor classification stage III B IPS 5) [12,13]. The case stands out due to medical management challenges and specific particularities (Table 1).

## 3. Discussion

### 3.1. HIV and COVID-19

The susceptibility and severity of COVID-19 in relation to HIV co-infection remain controversial. The risk of “cytokine storm” may be lower in PLWH with decreased CD4 lymphocytes, and without antiretroviral treatment. However, the lymphopenia associated with severe COVID-19, when combined with HIV-related lymphopenia, could explain the delayed clearance of SARS-CoV-2 [18,19]. Intravascular neutrophil extracellular trap (NET) capture is a defense mechanism used by both HIV and SARS-CoV-2, which may be aberrantly expressed [20].

A meta-analysis of 22 studies, including over 20 million PLWH, demonstrated a significantly higher risk of SARS-CoV-2 infection and related mortality, compared with HIV-negative individuals. Chronic inflammation associated with persistent HIV infection may be exacerbated by the massive release of proinflammatory cytokines during severe COVID-19. Additionally, HIV-related chronic inflammation acts as a procoagulant factor, which could be superposed to hypercoagulability induced by SARS-CoV-2 infection and contribute to severe complications and increased mortality in PLWH [21]. The potential benefits of certain antiretrovirals, such as tenofovir or protease inhibitors, in COVID-19 treatment have not been proven [22].

A study published in *AIDS* in November 2024 assessed the impact of the COVID-19 pandemic on HIV outcomes in the United States. The study found a 4.4% reduction in HIV incidence during 2020–2021, attributed to decreased transmission behaviors, despite disruptions in prevention and care services. However, HIV incidence increased by 2% from 2022 to 2024 due to less effective viral suppression among people living with HIV, suggesting that a continuous increase in incidence is expected post-pandemic [23].

### 3.2. Hodgkin Lymphoma and COVID-19

A study from two centers in Wuhan, China, involving 128 hospitalized patients with onco-hematological diseases showed that hematological malignancy does not significantly influence the rate of COVID-19 infection, but increases its severity and fatality, compared to patients with non-hematological malignancy [24].

A multicenter retrospective study from 19 centers in Madrid, Spain, evaluating 177 adult patients with COVID-19 and lymphoma, including diffuse large B-cell lymphoma, follicular lymphoma, other aggressive lymphomas (such as mantle cell lymphoma, primary effusion lymphoma, and natural killer—cell and T-cell neoplasms), other indolent lymphomas (such as marginal zone lymphoma, mucosa-associated lymphoid tissue lymphoma, lymphoplasmacytic lymphoma, and hairy cell leukemia), and HL. The overall mortality rate was 34.5%, regardless of the therapeutic regimens [25].

Another study on 3801 cases with lymphoproliferative and myeloproliferative malignancies developed 63.8% severe or critical COVID-19, and the overall mortality rate was 31.2%. COVID-19 was the primary cause of death in 58.1% of the cases, while both COVID-19 and hematological malignancies contributed to death in 13.1% of the cases, highlighting the high risk of lethal complications [26].

Several reported case studies have analyzed different aspects of the association between Hodgkin lymphoma and COVID-19, such as the risk of neoplastic lymphopenia and the secondary effects of immunosuppressive therapies on the severe progression of COVID-19, the suboptimal humoral response post-infection and post-vaccination for long-term immune protection, and the risk of reinfections, as well as the benefits of using monoclonal antibodies (pembrolizumab) in increasing the survival rate of patients with classical Hodgkin lymphoma. It was hypothesized that COVID-19 could trigger the appearance of lymphomas in patients with predisposing diseases, but extensive studies are required. The molecular mechanisms of certain coronaviruses in inducing cellular apoptosis and their influence on the progression of lymphoproliferative diseases are unclear [27,28,29,30].

### 3.3. HIV and Cancer

Human immunodeficiency virus (HIV) infection leads to progressive immunosuppression, increasing the risk of various cancers, including lymphomas [31]. The oncogenic mechanisms associated with HIV include direct effects of the virus, co-infections with other oncogenic viruses, and dysregulated cytokine levels that promote lymphoma development [32].

HL, although not classified as an AIDS-defining cancer, occurs at a higher incidence in people living with HIV. Due to antiretroviral treatment, the incidence of AIDS-related diseases, such as non-HL, has decreased, whereas HL has remained stable [12,33].

A recent meta-analysis found that the risk of developing HL in people living with HIV (PLWH) is 5 to 26 times higher than in the general population, while the prognosis of HL is similar in PLWH and the general population. Combined antiretroviral treatment with concomitant chemotherapy is highly recommended in PLWH with HL [34].

The antiretroviral regimen with tenofovir/emtricitabine/darunavir/cobicistate was highly recommended by previous EACS guidelines as a first-line therapy for treatment-naïve HIV patients, but drug interactions with oncologic drugs should be considered. Concomitant chemotherapy requires a review of antiretroviral therapy, with preferable present options being bictegravir/emtricitabine/tenofovir alafenamide or Dolutegravir/Lamivudine [35,36].

According to National Comprehensive Cancer Network (NCCN) Guidelines from November 2021, the optimal therapeutic combinations should be ABVD (Adrymicin/Bleomycin/Vinblastine/Dacarbazine) or Brentuximab + AVD (Adrymicin/Vinblastine/Dacarbazine) [16]. However, the administration of Doxorubicin, Vinblastine, and Brentuximab should be limited in cases of cholestasis with total bilirubin levels exceeding 5 mg/dL, which was the case for our patient [26,37].

### 3.4. Immunophenotypic Profiling of Lymphoma in Patients with HIV

Large cells exhibited CD30 positivity, while rare cells were CD20-positive and showed a high Ki-67 proliferation index, with negative staining for CD3. CD3 positivity was observed in typical small lymphocytes and activated T cells, one of which was PD-1-positive. The large cells also expressed PAX5 and were negative for CD15, supporting the diagnosis of mixed-cellularity classical HL.

The immunohistochemical findings provide a detailed characterization of mixed-cellularity classical HL (MCCHL) in the context of HIV infection. CD30 positivity in large cells is a characteristic marker of Reed–Sternberg cells, confirming their presence and supporting the diagnosis of classical HL. PAX5 expression, alongside the absence of CD15, is consistent with a classical HL subtype but with an atypical immunophenotype. While CD15 negativity is less common, it has been described in certain cases of MCCHL, particularly in immunocompromised patients [38].

Rare CD20-positive cells suggest partial retention of B-cell markers, a feature occasionally observed in HIV-associated HL, possibly reflecting the incomplete transformation of Reed–Sternberg cells [39]. Ki-67 positivity in large cells indicates a high proliferative index, correlating with the aggressive clinical course often seen in HIV-associated lymphomas. This elevated Ki-67 index suggests a rapidly proliferating tumor, emphasizing the need for timely and aggressive treatment [40].

Our case highlights the profound interplay between HIV-related immunosuppression and lymphomagenesis. Chronic immune activation and loss of immune surveillance allow the reactivation of latent viruses, such as the Epstein–Barr virus (EBV), which is frequently implicated in HL among HIV-positive patients [41,42]. The altered cytokine environment in HIV, characterized by elevated levels of IL-6 and other proinflammatory cytokines, creates a microenvironment that facilitates malignant transformation [43].

The atypical immunohistochemical profile, including CD15 negativity, may reflect the unique biology of lymphomas in immunosuppressed hosts, suggesting potential viral or host-related influences on tumor evolution [44]. The immunohistochemical findings offer valuable insights into the tumor microenvironment, particularly in the context of HIV. The presence of CD3-positive small lymphocytes, coupled with PD-1 expression, highlights the intense inflammatory nature of HL. Furthermore, PD-1 positivity suggests the presence of exhausted T cells, a hallmark of both HIV-related immune dysfunction and the immune-evasive mechanisms employed by HL [45].

HIV complicates the interpretation of immunohistochemical findings, particularly in HL, where the reactive immune infiltrate can resemble other lymphoproliferative disorders, such as diffuse large B-cell lymphoma or EBV-positive mucocutaneous ulcer. Accurate diagnosis requires a thorough integration of clinical, histopathological, and molecular data, especially in immunocompromised patients with atypical presentations [46].

To summarize, immunohistochemistry (IHC) is a complex diagnostic method essential for therapeutic decisions in HL. However, interpretative errors can occur, as observed in the initial biopsy of our case. The accuracy and reliability of results are influenced by factors such as microanatomic distribution and staining intensity, which can be affected by errors like nonspecific staining, tissue artifacts, inadequate inactivation of endogenous peroxidase activity, and cross-reactivity. To minimize these errors and improve the interpretation of IHC results, standardizing laboratory procedures and continuous training of diagnostic personnel are essential. Implementing strict protocols and using appropriate controls can help reduce variability and enhance the reliability of immunohistochemical diagnosis in HL [47,48]. The selection of monoclonal antibody panels for tissue protein identification can influence diagnostic accuracy, as no single marker has 100% sensitivity for a specific tumor. Standardizing laboratory procedures, implementing appropriate controls, enhancing staff expertise, and adopting digital technologies can improve the reliability of immunohistochemical diagnosis in Hodgkin lymphoma [49].

### 3.5. COVID-19 Vaccination in People with HIV or Lymphoma

COVID-19 vaccines are generally well tolerated and effective in most individuals with HIV infection. Depending on the severity of immunosuppression, the risk of severe and prolonged disease increases in unvaccinated individuals, and vaccine efficacy may be lower, with breakthrough infections and a more rapid decline in vaccine-induced protection observed, likely due to a suboptimal humoral and cellular immune response [50].

An insufficient humoral and cellular response to vaccination has also been demonstrated in patients with lymphoma, but the safety of vaccine administration has been confirmed even during active antineoplastic treatment. Variations in vaccine response depended on the time interval between chemotherapy completion and vaccination, the serum concentration of IgM and IgG, and the proportion of CD19- and CD4-positive cells in peripheral blood [51].

COVID-19 vaccination guidelines recommend prioritizing vaccination and administering additional booster doses for both individuals with HIV and those with lymphoma, as well as closely monitoring these populations. In the future, further studies are needed on vaccination regimens, dosing intervals, immune responses after the administration of updated vaccines for new viral variants, and the combination of vaccination with anti-COVID-19 therapies or other prophylactic measures [52].

## 4. Conclusions

Limited access to medical facilities during the COVID-19 pandemic delayed the patient’s evaluation and treatment, exacerbating the progression of HIV-associated lymphoma. Interpretative errors in immunohistochemistry (IHC) are possible in Hodgkin lymphoma (HL) among HIV patients. A late HIV diagnosis indicates low efficiency of education and screening programs, which declined during the COVID-19 pandemic. The late diagnosis of HIV infection, advanced Hodgkin lymphoma (HL), and concurrent COVID-19 form a complex pathogenic triad that is challenging to manage and is associated with severe outcomes and increased mortality.

## Figures and Tables

**Table 1:** Peculiarities of the case.

Three severe overlapping conditions were concomitantly diagnosed: AIDS, HL, and COVID-19. MSOF was the consequence of severe COVID-19, including liver failure, respiratory failure, hematological, and metabolic dysfunctions. Evaluation of multiple poorly defined opaque images in both lungs described as “ground glass” in the COVID-19 context required the consideration of a differential diagnosis including pneumocystosis, tuberculosis, or lymphomatous pneumonia. Limited access to healthcare during the COVID-19 pandemic delayed the patient’s presentation for hematological investigations, but COVID-19 was a healthcare-associated infection. Liver injury could be related to COVID-19, as well as to lymphomas or an adverse drug event. Discordant results of the first IHC and HP examinations were a possible error related to the use of an insufficient panel of immunostains that cause misdiagnosis and delayed onco-hematological treatment [14,15]. HL is a non-AIDS-related malignancy and was diagnosed at an advanced stage, with a poor prognosis according to the International Prognostic Score [16,17]. Late HIV presentation might reflect the insufficient preventive education and HIV screening in rural areas. Earlier HIV diagnosis and care could have been the “lifeline anchor” for our patient. COVID-19 vaccination could help prevent the fatal progression of newly diagnosed HIV and Hodgkin lymphoma.

## Data Availability

Data regarding the findings are available within the manuscript.

## Abbreviations

The following abbreviations are used in this manuscript:

AIDS	Acquired immunodeficiency syndrome
ALT	Alanin aminotransferase
ARN-HIV	HIV viral load
AST	Aspartate aminotransferase
BP	Blood pressure
CBC	Complete blood count
COVID-19	Coronavirus Disease 2019
CT	Computed tomography
ECOG	Eastern Cooperative Oncology Group
ELISA	Enzyme-linked immunosorbent assay
GSC	Glasgow scale coma
HL	Hodgkin’s lymphoma
HIV	Human immunodeficiency virus
HBc Ab	Hepatitis B core antibody
HBs-Ag	Hepatitis B surface antigen
HCV-Ab	Hepatitis C virus antibody
HP	Histopathology
HR	Heart rate
IHC	Immunohistochemy
NET	Neutrophil extracellular trap
PLWH	People living with HIV
RT-PCR	Real-time polymerase chain reaction
SARS-CoV-2	Severe Acute Respiratory Syndrome Coronavirus 2
SO2	Saturation of oxygen

## Appendix A

**Table A1:** The dynamic of laboratory tests.

Test Name	Week 0	Week 2	Week 3	Week 4	Week 6	Week 8	Week 9
WBC ×10^9^ (4–9)	*3.8*	*3.3*	*4.2*	*3.7*	*1.5*	*1.7*	*1.5*
N ×10^6^ (2000–6300)	2150	*1370*	2610	2990	*1140*	*1300*	*1340*
CD4/mmc (430–1800)	*112*		*73*	*46*	*21*		
Hb g/L (130–170)	*111*	*97*	*106*	*73*	*63*	*83*	*66*
PTL ×10^9^ (150–450)	*117*	*139*	155.6	*111*	*47*	*21*	*4*
CRP mg/L (0–10)	6.16	*42.9*	*109.1*	*114.9*	*65.1*	*113.9*	*115.9*
ERS mm/h (0–20)	*80*	*110*	*110*	*100*	*120*	*56*	*34*
Cr. mg/dL (0.7–1.3)	0.76	0.68	1.36	0.71	0.76	0.53	0.52
ALT U/L (0–40)	*84*	32.4	*185.7*	*58.3*	32.5	*113.2*	*135.4*
Bt mg/dL (0.3–1.2)		0.91	*1,24*	*8.2*	*12.4*		*21.5*
Albumin g/L (38–54)	*26.4*			*16.4*	*18.6*	*21.1*	*33.3*
D-Drs ug/L (˂500)	*4616*	*2445*	*1999.9*	*2075*	*940*	*1007*	*632*
LA mg/dL (5–20)	18.28	*24.13*	*23.9*	*31.7*		18.7	*38.2*
SARS-CoV-2	*positive*		*positive*		*positive*		*positive*
HCV-Ab	negative						
HBsAg HBcAb	negative negative						
Tumoral markers	Safety limits CA 19.9 = 21 U/mL (1.2–30.9), CEA = 0.5 ng/mL (0.5–5), PSA = −0.19 (0–4 ng/mL), AFP = 0.5 ng/mL (1.3–8.1)						
Bone marrow aspiration	Erythrocyte series: normal percentage; erythroid precursors with moderate dysplastic appearance. Granulocyte series: increased percentage; granulocyte precursors with dysplastic appearance. Lymphoplasmocyte series: decrease percentage. Platelet series: normal; megakaryocytes with dysplastic appearance.						

Legend: ALT: alaninaminotranspherase; Bt: total bilirubin; Cr.: creatinine; CRP: C-reactive protein; D-Drs: D-dimers; ERS: erytrocite sedimentation rate; Hb: hemoglobin; HBs-Ag; surface antigen of the hepatitis B virus; HBc-Ab: antibodies against the core antigen of hepatitis B; HCV-Ab: antibodies of the hepatitis C virus; LA: lactic acid; N: neutrophyle; PTL: platelet; WBC: white blood cell.

## References

[1] Alfano V., Ercolano S. (2020). The Efficacy of Lockdown Against COVID-19: A Cross-Country Panel Analysis. Appl. Health Econ. Health Policy.

[2] De Vincentiis L., Carr R.A., Mariani M.P., Ferrara G. (2021). Cancer diagnostic rates during the 2020 ‘lockdown’, due to COVID-19 pandemic, compared with the 2018-2019: An audit study from cellular pathology. J. Clin. Pathol..

[3] Maringe C., Spicer J., Morris M., Purushotham A., Nolte E., Sullivan R., Rachet B., Aggarwal A. (2020). The impact of the COVID-19 pandemic on cancer deaths due to delays in diagnosis in England, UK: A national, population-based, modelling study. Lancet Oncol..

[4] Siegler J.E., Heslin M.E., Thau L., Smith A., Jovin T.G. (2020). Falling stroke rates during COVID-19 pandemic at a comprehensive stroke center. J. Stroke Cerebrovasc. Dis..

[5] Colivicchi F., Di Fusco S.A., Magnanti M., Cipriani M., Imperoli G. (2020). The Impact of the Coronavirus Disease-2019 Pandemic and Italian Lockdown Measures on Clinical Presentation and Management of Acute Heart Failure. J. Card. Fail..

[6] Nopp S., Janata-Schwatczek K., Prosch H., Shulym I., Königsbrügge O., Pabinger I., Ay C. (2020). Pulmonary embolism during the COVID-19 pandemic: Decline in diagnostic procedures and incidence at a university hospital. Res. Pract. Thromb. Haemost..

[7] Darcis G., Vaira D., Moutschen M. (2020). Impact of coronavirus pandemic and containment measures on HIV diagnosis. Epidemiol. Infect..

[8] Arbune M., Padurariu-Covit M.D., Tiutiuca C., Mihailov R., Niculet E., Arbune A.A., Tatu A.L. (2024). Unusual Localization of AIDS-Related Kaposi’s Sarcoma in a Heterosexual Male during the COVID-19 Pandemic: A Case Report. Trop. Med. Infect. Dis..

[9] Gaddey H.L., Riegel A.M. (2016). Unexplained Lymphadenopathy: Evaluation and Differential Diagnosis. Am. Fam. Physician.

[10] Oken M.M., Creech R.H., Tormey D.C., Horton J., Davis T.E., McFadden E.T., Carbone P.P. (1982). Toxicity and response criteria of the Eastern Cooperative Oncology Group. Am. J. Clin. Oncol..

[11] Xu W., Gu B., Lotter W.E., Kehl K.L. (2024). Extraction and Imputation of Eastern Cooperative Oncology Group Performance Status From Unstructured Oncology Notes Using Language Models. JCO Clin. Cancer Inform..

[12] CDC (1994). 1994 Revised classification system for human immunodeficiency virus infection in children less than 13 years of age. Morb. Mortal. Wkly. Rep..

[13] Lister T.A., Crowther D., Sutcliffe S.B., Glatstein E., Canellos G.P., Young R.C., Rosenberg S.A., Coltman C.A., Tubiana M. (1989). Report of a committee convened to discuss the evaluation and staging of patients with Hodgkin’s disease: Cotswolds meeting. J. Clin. Oncol..

[14] Shi Y., Mi L., Lai Y., Zhao M., Jia L., Du T., Song Y., Li X. (2023). PD-L1 immunohistochemistry assay optimization to provide more comprehensive pathological information in classic Hodgkin lymphoma. J. Hematop..

[15] Li X. (2015). Pitfalls in the pathological diagnosis of lymphoma. Chin. Clin. Oncol..

[16] Hoppe R.T., Advani R.H., Ai W.Z., Ambinder R.F., Armand P., Bello C.M., Benitez C.M., Chen W., Dabaja B., Daly M.E. (2022). NCCN Guidelines^®^ Insights: Hodgkin Lymphoma, Version 2.2022. J. Natl. Compr. Cancer Netw..

[17] Hasenclever D., Diehl V. (1998). A prognostic score for advanced Hodgkin’s disease. International Prognostic Factors Project on Advanced Hodgkin’s Disease. N. Engl. J. Med..

[18] Noy A. (2019). Optimizing treatment of HIV-associated lymphoma. Blood.

[19] Mehta P., McAuley D.F., Brown M., Sanchez E., Tattersall R.S., Manson J.J., HLH Across Speciality Collaboration U.K. (2020). COVID-19: Consider cytokine storm syndromes and immunosuppression. Lancet.

[20] Mozzini C., Girelli D. (2020). The role of Neutrophil Extracellular Traps in COVID-19: Only an hypothesis or a potential new field of research?. Thromb. Res..

[21] Tesoriero J.M., Swain C.E., Pierce J.L., Zamboni L., Wu M., Holtgrave D.R., Gonzalez C.J., Udo T., Morne J.E., Hart-Malloy R. (2021). COVID-19 Outcomes Among Persons Living With or Without Diagnosed HIV Infection in New York State. JAMA Netw. Open.

[22] Ssentongo P., Heilbrunn E.S., Ssentongo A.E., Advani S., Chinchilli V.M., Nunez J.J., Du P. (2021). Epidemiology and outcomes of COVID-19 in HIV-infected individuals: A systematic review and meta-analysis. Sci. Rep..

[23] Viguerie A., Jacobson E.U., Hicks K.A., Bates L., Carrico J., Honeycutt A., Lyles C., Farnham P.G. (2024). Assessing the Impact of COVID-19 on HIV Outcomes in the United States: A Modeling Study. Sex. Transm. Dis..

[24] He W., Chen L., Yuan G., Fang Y., Chen W., Wu D., Liang B., Lu X., Ma Y., Li L. (2020). COVID-19 in persons with haematological cancers. Leukemia.

[25] Regalado-Artamendi I., Jiménez-Ubieto A., Hernández-Rivas J., Navarro B., Núñez L., Alaez C., Córdoba R., Peñalver F.J., Cannata J., Estival P. (2021). Risk Factors and Mortality of COVID-19 in Patients with Lymphoma: A Multicenter Study. Hemasphere.

[26] Pagano L., Salmanton-García J., Marchesi F., Busca A., Corradini P., Hoenigl M., Klimko N., Koehler P., Pagliuca A., Passamonti F. (2021). COVID-19 infection in adult patients with hematological malignancies: A European Hematology Association Survey (EPICOVIDEHA). J. Hematol. Oncol..

[27] Yonal-Hindilerden I., Hindilerden F., Mastanzade M., Tiryaki T.O., Tasan-Yenigun S., Bilen Y., Aksoz S., Cagatay A.A., Nalcaci M. (2021). Case Report: Severe COVID-19 Pneumonia in a Patient With Relapsed/Refractory Hodgkin’s Lymphoma. Front. Oncol..

[28] Fakharian A., Ebrahimibagha H., Mirenayat M.S., Farahmandi F. (2021). COVID-19 Reinfection in a Patient with Hodgkin Lymphoma: A Case Report. Tanaffos.

[29] Hamed M., Alamoudi D. (2023). Recurrent COVID-19 Infection in a Refractory/Classical Hodgkin’s Lymphoma Patient Undergoing Autologous Stem Cell Transplantation: A Case Report. Cureus.

[30] Cartas U.S., González J.L.V., Hernandez W., Ríos C.A.G. (2022). Lymphoma as a Complication of Recurrent COVID-19 Infection in Patients with Rheumatic Disease. Ann. Case Rep..

[31] Veyri M., Lavolé A., Choquet S., Costagliola D., Solas C., Katlama C., Poizot-Martin I., Spano J.P. (2021). Do people living with HIV face more secondary cancers than general population: From the French CANCERVIH network. Bull. Cancer.

[32] Berhan A., Bayleyegn B., Getaneh Z. (2022). HIV/AIDS Associated Lymphoma: Review. Blood Lymphat. Cancer.

[33] Dolcetti R., Gloghini A., Caruso A., Carbone A. (2016). A lymphomagenic role for HIV beyond immune suppression?. Blood.

[34] Navarro J.T., Moltó J., Tapia G., Ribera J.M. (2021). Hodgkin Lymphoma in People Living with HIV. Cancers.

[35] European AIDS Clinical Society (EACS) Guideline Version 12.0 October 2023. https://www.eacsociety.org/media/guidelines-12.0.pdf.

[36] Branch C., Parson-Martinez J., Cory T.J. (2025). Drug-drug interactions in HIV-infected patients receiving chemotherapy. Expert. Opin. Drug Metab. Toxicol..

[37] Zelenetz A.D., Gordon L.I., Chang J.E., Christian B., Abramson J.S., Advani R.H., Bartlett N.L., Budde L.E., Caimi P.F., De Vos S. (2021). NCCN Guidelines^®^ Insights: B-Cell Lymphomas, Version 5.2021. J. Natl. Compr. Cancer Netw..

[38] Konkay K., Paul T.R., Uppin S.G., Rao D.R. (2016). Hodgkin lymphoma: A clinicopathological and immunophenotypic study. Indian J. Med. Paediatr. Oncol..

[39] Liu Y., Xie X., Li J., Xiao Q., He S., Fu H., Zhang X. (2024). Immune Characteristics and Immunotherapy of HIV-Associated Lymphoma. Curr. Issues Mol. Biol..

[40] Mao X., Li Y., Liu S., He C., Yi S., Kuang D., Xiao M., Zhu L., Wang C. (2023). Multicolor flow cytometric assessment of Ki67 expression and its diagnostic value in mature B-cell neoplasms. Front. Oncol..

[41] Verdu-Bou M., Tapia G., Hernandez-Rodriguez A., Navarro J.T. (2021). Clinical and Therapeutic Implications of Epstein-Barr Virus in HIV-Related Lymphomas. Cancers.

[42] Muncunill J., Baptista M.J., Hernandez-Rodríguez Á., Dalmau J., Garcia O., Tapia G., Moreno M., Sancho J.M., Martínez-Picado J., Feliu E. (2019). Plasma Epstein-Barr Virus Load as an Early Biomarker and Prognostic Factor of Human Immunodeficiency Virus-related Lymphomas. Clin. Infect. Dis..

[43] Grebenciucova E., VanHaerents S. (2023). Interleukin 6: At the interface of human health and disease. Front. Immunol..

[44] Ganapathi K.A., Nicolae A., Egan C., Geng H., Xi L., Pack S.D., McFadden J.R., Raffeld M., Jaffe E.S., Pittaluga S. (2024). Peripheral T-cell lymphomas expressing CD30 and CD15 expand the spectrum of anaplastic large cell lymphoma, ALK-negative. Br. J. Haematol..

[45] Zhao H., Cai S., Xiao Y., Xia M., Chen H., Xie Z., Tang X., He H., Peng J., Chen J. (2024). Expression and prognostic significance of the PD-1/PD-L1 pathway in AIDS-related non-Hodgkin lymphoma. Cancer Med..

[46] Hübel K., Bower M., Aurer I., Bastos-Oreiro M., Besson C., Brunnberg U., Cattaneo C., Collins S., Cwynarski K., Pria A.D. (2024). Human immunodeficiency virus-associated Lymphomas: EHA-ESMO Clinical Practice Guideline for diagnosis, treatment and follow-up. Hemasphere.

[47] Mebratie D.Y., Dagnaw G.G. (2024). Review of immunohistochemistry techniques: Applications, current status, and future perspectives. Semin. Diagn. Pathol..

[48] Magaki S., Hojat S.A., Wei B., So A., Yong W.H. (2019). An Introduction to the Performance of Immunohistochemistry. Methods Mol. Biol..

[49] Yimpak P., Bumroongkit K., Tantiworawit A., Rattanathammethee T., Aungsuchawan S., Daroontum T. (2024). Immunohistochemistry-based investigation of MYC, BCL2, and Ki-67 protein expression and their clinical impact in diffuse large B-cell lymphoma in upper Northern Thailand. PLoS ONE.

[50] Höft M.A., Burgers W.A., Riou C. (2024). The immune response to SARS-CoV-2 in people with HIV. Cell Mol. Immunol..

[51] Narita K., Nakaji S., Tabata R., Terao T., Kuzume A., Tsushima T., Ikeda D., Fukumoto A., Miura D., Takeuchi M. (2022). Antibody response to COVID-19 vaccination in patients with lymphoma. Int. J. Hematol..

[52] Hall V.G., Teh B.W. (2023). COVID-19 Vaccination in Patients with Cancer and Patients Receiving HSCT or CAR-T Therapy: Immune Response, Real-World Effectiveness, and Implications for the Future. J. Infect. Dis..

